# Testing ACL-Reconstructed Football Players on the Field: An Algorithm to Assess Cutting Biomechanics Injury Risk Through Wearable Sensors

**DOI:** 10.3390/sports13110391

**Published:** 2025-11-05

**Authors:** Stefano Di Paolo, Marianna Viotto, Margherita Mendicino, Chiara Valastro, Alberto Grassi, Stefano Zaffagnini

**Affiliations:** 1 2nd Orthopaedic and Traumatologic Clinic, IRCCS Istituto Ortopedico Rizzoli, 40136 Bologna, Italy; margherita.mendicino@ior.it (M.M.); chia.valastro@gmail.com (C.V.); alberto.grassi@ior.it (A.G.); stefano.zaffagnini@unibo.it (S.Z.); 2 Pediatric Orthopedic and Traumatology, IRCCS Istituto Ortopedico Rizzoli, 40136 Bologna, Italy; marianna.viotto@ior.it; 3 Department of Biomedical and Neuromotor Sciences, University of Bologna, 40123 Bologna, Italy

**Keywords:** ACL, biomechanics, wearables inertial sensors, return to sport, ecological dynamics, change of direction

## Abstract

Anterior cruciate ligament (ACL) injuries in football mostly occur during defensive (pressing) cut maneuvers. Football-specific cutting movements are key to identifying dangerous biomechanics but hard to evaluate clinically. This study aimed to develop a practical field-based tool—Anterior Cruciate Ligament Injury Risk Profile Detection (ACL-IRD)—to assess ACL injury risk during return to sport (RTS). It was hypothesized that the ACL-IRD could detect ACL injury risk profiles after ACLR players had RTS clearance. Sixty-one footballers (21 ACLR, 40 healthy; 16.2 ± 2.2 years old, >14 months post-surgery) were tested on a regular football pitch. Players performed pre-planned (AGTT) and unplanned football-specific cut maneuvers simulating defensive pressing (FS deceiving action). Kinematic data were collected via eight wearable inertial sensors (MTw Awinda, Movella) on trunk and lower limbs. The ACL-IRD analyzed biomechanics in three risk categories, knee valgus collapse, sagittal knee loading, and trunk–pelvis imbalance, using thresholds from healthy players. A clinician-friendly, automatic report was generated. At-risk biomechanics were identified in 36–37/104 AGTT trials and 25–41/97 FS deceiving actions (at initial contact and peak knee flexion). Over 60% of risky trials involved the ACLR limb. Major risk factors were altered knee/hip flexion ratio, knee valgus, and hip abduction. The ACL-IRD is a novel, clinical-friendly tool designed to identify potential ACL injury risk profiles and is intended to support safer RTS decisions.

## 1. Introduction

Anterior cruciate ligament (ACL) injury rates are worryingly rising in pediatric populations [1,2]. The surgical and rehabilitation path of a skeletally immature patient is even more complex than an adult one [3,4], and the criteria for safely returning to sports (RTS) after pediatric ACL reconstruction (ACLR) are not well-defined [5,6]. Up to 35% of young patients experience a second ACL injury, either as a retear or damage to the opposite knee [5,7]. This rate could even worsen when an ACL injury happens with a non-contact mechanism, according to UEFA studies [8]. With youth sports becoming more demanding and competitive, incorporating rigorous RTS testing has become crucial to ensure athletes meet necessary milestones before clearance to play [9].

Movement biomechanics has become crucial in the rehabilitation and RTS after ACLR. Recent studies have identified clear injury risk patterns associated with non-contact ACL injury in football [8,10,11,12]. It has been suggested that ACL rupture results from a combination of multiple biomechanical factors, a so-called “perfect ACL loading storm” [10,13,14,15,16]. These patterns involve whole-body mechanics and are likely to occur during cutting movements, such as the ones occurring during pressing, deceiving actions, and decelerations, which could be barely reproduced in a laboratory environment. Data collected in ecological environments has therefore been advocated to improve injury risk pattern detection fidelity [17,18,19,20,21].

In this scenario, dedicated algorithms to test football-specific cut maneuver tasks captured on the field become crucial to detecting such dangerous biomechanical patterns. Field-based assessments preserve the player–environment interaction, providing more accurate data on movement patterns and high-risk biomechanics. Such insights have the potential to reshape prevention programs by identifying at-risk players and tailoring data-driven interventions [9,22]. However, translating this information into everyday clinical practice remains a significant challenge.

The aim of the present study was to provide a practical tool to assess the ACL injury risk during the RTS continuum through sport-specific biomechanical testing. The tool adopts a dedicated algorithm that was named “Anterior Cruciate Ligament Injury Risk Profile Detection” (ACL-IRD). It was hypothesized that the ACL-IRD algorithm would be able to detect risk factors for ACL injury even after clearance for RTS in ACLR players. The ultimate goal of this tool is to convert complex multifaceted biomechanical data into practical insights for healthcare professionals, including surgeons and physiotherapists, supporting them in the RTS decision-making continuum through real-time reporting feedback based on objective measures of players’ motion.

## 2. Materials and Methods

### 2.1. Participants

Sixty-one young competitive football (soccer) players were prospectively enrolled in the study. Out of these, 21 players (16.8 ± 1.6 years, 15 males and 6 females) had undergone ACLR through pediatric technique [4] and had been cleared for RTS after standard of care rehabilitation (12.0 ± 7.3 months after surgery); 40 players matched in age and level (15.9 ± 2.4 years, 24 males and 16 females) were included as a healthy control group. All ACLR players were operated on by the same experienced surgeon (S.Z). Inclusion criteria were age below 18 years old, being an outfield player (no goalkeepers), and having undergone a non-contact ACL injury during sport activities (for ACLR patients only). Exclusion criteria were cardiopulmonary diseases, other severe musculoskeletal injuries in career (>28 days out), inability to perform the required movement tasks, and not being cleared for RTS (for ACLR patients only). Healthy control players were free from severe musculoskeletal injuries (>28 days stop) at the time of and for the first year after data collection. Written informed consent for participation was obtained from all players prior to inclusion in the study. For participants under 18 years old, both the written consent of a parent/legal guardian and the player’s assent were obtained. The study was approved by the Bioethical Committee of the University of Bologna (IRB approval: n. 25861 of 10 February 2020) in accordance with the Declaration of Helsinki.

### 2.2. Data Collection

Data collection was held on a football pitch with artificial turf, under daily sunny conditions, during the competitive phase of the season, to minimize potential biases related to player and surface conditions. Before starting with data acquisition, anthropometric measures were collected for each player, followed by a warm-up performed autonomously by all athletes (5–10 min) and familiarization trials (at least two per test, not recorded). Two tasks were selected to simulate cutting maneuvers. In football, these movements are widely recognized as situational patterns associated with non-contact ACL injuries. Therefore, they require close consideration for identifying deficits that may increase the risk of re-injury and for defining targeted interventions to ensure a safe RTS [14,23,24].

In this regard, every athlete performed two high-dynamic tasks: 90° change of direction within the Agility *T*-test (AGTT) and unplanned football-specific change of direction (FS deceiving action) [25,26]. For each task, two to three repetitions per side (left and right) were recorded for each player to ensure consistency and reliability of the data. A rest of 1–2 min in between the trials and 3–5 min in between the two tasks was given to the players. AGTT is widely used in multidirectional sports to evaluate an athlete’s agility performance, including their ability to accelerate, decelerate, and change direction efficiently [27,28]. The test consists of five sequential phases: a straight-line sprint from a standing start to a central cone, a lateral shuffle to one side (pre-determined and communicated to the athlete), a lateral shuffle to the opposite side, a return shuffle to the central cone and a backward sprint to the starting position. Athletes were instructed to maintain consistent orientation relative to their initial starting position, and they were pushed to sprint at their maximum and complete the drill in the shortest time possible. The test did not include any other player/element. On the other hand, the FS deceiving action evaluates an athlete’s ability to react to an unplanned directional change, simulating the dynamic demands of a real football game. In this test, athletes run in a straight line before responding to an opponent player in ball possession. They were not told which direction the opponent would choose to change. The opponent stood still, 2 m away from the cones, and only changed direction when the player being tested was approaching the cone, simulating a deceptive action and requiring an immediate change of direction (COD) [29].

A set of eight wearable inertial sensors (MTw Awinda, Xsens Technologies, Enschede, The Netherlands) placed on the trunk (C7), pelvis (L5), and lower limbs was used to capture motion during the execution of the tasks [30,31,32]. The full-body joint kinematics was collected at a sampling frequency of 100 Hz. The sensor placement was performed by a single experienced operator. The system calibration was performed in both static (upright standing) and dynamic (walking) conditions per athlete. The reliability of the wearable system in the assessment of high dynamic movements was confirmed in previous studies [30,31].

### 2.3. ACL-IRD Algorithm

#### 2.3.1. ACL-IRD Algorithm—Overview

**Figure 1:**
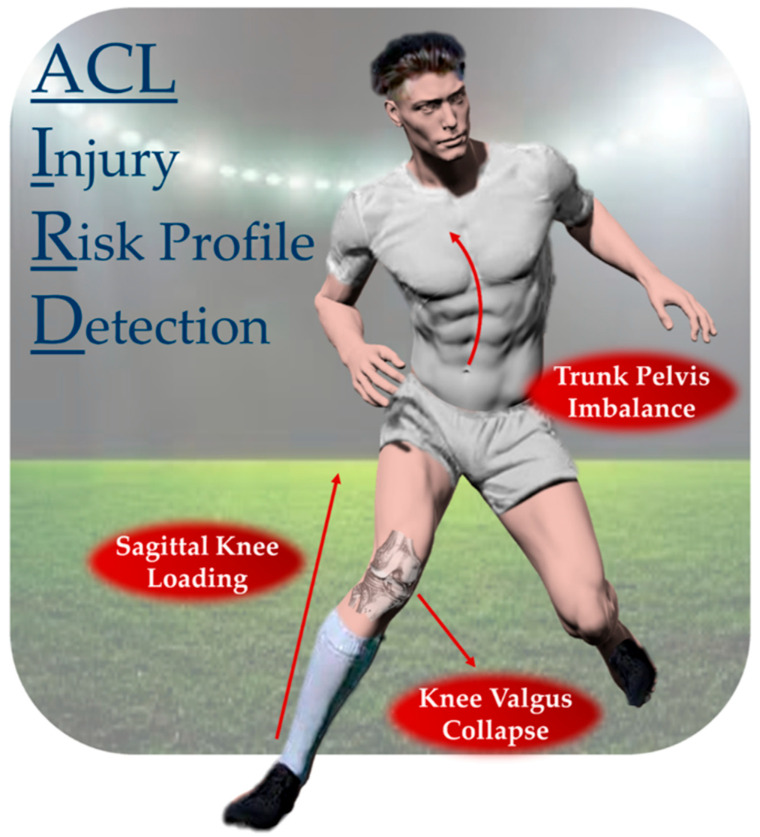
Main risk categories for non-contact ACL injury in football players. The three categories were decomposed into nine biomechanical risk factors in the ACL-IRD algorithm. The simultaneous detection of the risk factors determines a trial “at risk”.

The data extracted from the wearable sensor system were processed in the proprietary wearable sensors software and in a custom Matlab script (vR2022a, The MathWorks, Natick, MA, USA) and were used to build the ACL-IRD algorithm. The goal of the ACL-IRD algorithm is to detect key features of the players’ movement biomechanics that would put them at risk of overloading the knee joint and increasing the risk of sustaining an ACL injury according to the current literature [10,13,14] (Figure 1). The algorithm detects the biomechanical risk profile of any cut maneuver performed by the player, considering key risk areas, positional and performance metrics (such as velocity and acceleration/decelerations) extracted from the cutting phase, intra-limb asymmetry, and the number of trials in which the player’s biomechanical patterns were classified as indicative of a risk profile. The benchmark data for ACLR players were provided by the matched healthy controls.

The ACL-IRD algorithm generates a patient-specific report, which is structured into six sections: (1) personal data, including the patient’s demographics and contextual information (injured leg, number of trials performed, etc.); (2) change of direction data (cut angle and duration); (3) performance data; (4) descriptive kinematics; (5) biomechanical risk factors analysis; and (6) final remarks. Each section is detailed in the paragraphs below.

Each section graphically displays the patient’s metrics relative to thresholds or intervals based on matched healthy controls’ reference data. This approach ensures a clear representation of how the patient’s metrics align or deviate from standard values, helping in the identification of potential risk factors. For both tests, the report maintains the same structure. Each test-specific section is divided into four key units. The detailed description of each section with the dedicated methodology, graphical representation, and source code are present in Supplementary Materials.

The ACL-IRD algorithm requires minimal computational power and Matlab software only (vR2022a, MathWorks, Natick, MA, USA). Clinicians simply need to run the report (by clicking the “Run” button in Matlab) and select the folder containing cut maneuver kinematics data as input; then, the report is automatically generated in real time.

#### 2.3.2. ACL-IRD Algorithm—Change of Direction Identification

At the beginning of the report, a brief explanation of the test is provided. Specific attention is given to the foot contact (FC) window, which is marked with a blue box in each figure illustrating the tasks (Online Resource S1, page 3, 12).

The FC window identifies the ultimate foot strike before COD [10]. This window is used as a primary focus for the evaluation of the cut angle, performance metrics, and kinematics [33,34]. According to the current literature, the cut angle significantly influences COD biomechanics and ACL injury risk [34]. The algorithm identifies the FC window and calculates the cut angle for each trial. The average cut angle across all trials is also reported.

The cut angle was determined using a multistep algorithm. Starting from the signal of pelvis vertical velocity, local minima were used to identify foot contacts throughout each trial, which enabled their segmentation into single steps [35,36]. For each step, the angle between the normalized vectors formed between the current step and the fifth step preceding and following it was iteratively calculated. After computing these angles, threshold filters were applied to exclude angles that were either excessively large or small. Specifically, angles below 10° and above 110° were discarded, as they could not plausibly represent cut angles associated with the two tasks performed by players. Among all the calculated cut angles that were maintained as reasonable values, the following additional criteria were applied to detect the most relevant cut angle for each trial: A first check on resultant pelvis velocity, in the mediolateral and anteroposterior directions, was performed by extracting local maxima and the subsequent minima, and angles in this interval were considered eligible as cut angles. To finalize the cut angle identification, the derivative of the mediolateral component of the pelvis velocity in a window succeeding the resultant velocity peak was analyzed. From this derivative signal, the first local maximum was identified, and only angles within a window of 500 ms around this maximum were considered eligible [10,37]. Finally, from eligible angles, the highest was taken as the final cut angle for the trial.

#### 2.3.3. ACL-IRD Algorithm—Performance

The players’ performance was evaluated for each trial and summarized in the Performance section of the report. Key velocity and acceleration features associated with changes of direction were extracted from the FC window identified. Previous studies have demonstrated that higher speeds and accelerations during COD increase the load on the ACL, thereby elevating the risk of injury [34,38].

Specifically, the following features were included: peak velocity, peak acceleration, minimum deceleration in a 2 s entry window before the foot stance (referred to as “IN” in Online Resource S1), and peak velocity and peak acceleration in the exit window after the foot stance (referred to as “OUT” in Online Resource S1).

**Figure 2:**
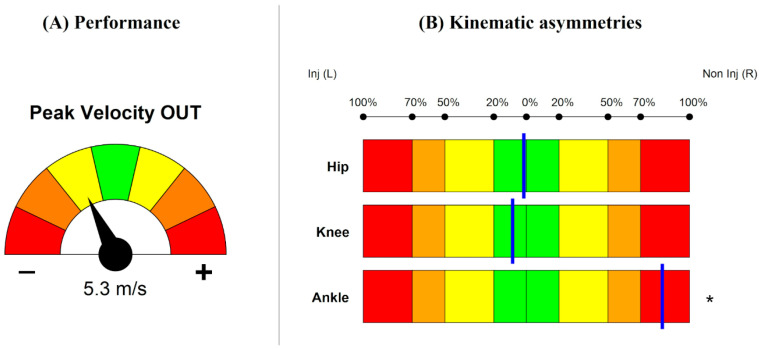
(**A**) Example of performance data visualization: speedometer for exit peak velocity. Green, yellow, orange, and red dials, respectively, represent z-score = 0, 1, 2, and 3 based on healthy controls reference; negative and positive needle orientation denote lower and higher performances than controls, respectively. (**B**) Example of Asymmetry Score for hip, knee, and ankle joints. Green: <20% asymmetry (AS = 0); yellow: 20–50% (AS = 1); orange: 50–70% (AS = 2); red: ≥70% (AS = 3). The blue line indicates the percentage of asymmetry between the limbs. Shifts to the left or right depend on which limb exhibits greater flexion values. An asterisk (*) denotes a significant deviation from normative values, corresponding to a z-score >3.

Players’ metrics were calculated relative to his/her center of mass as extracted from the wearable sensor system. For each performance metric, a color-coded speedometer visualization (Figure 2A) was employed to compare the measured value of each ACLR player in relation to normative data ranges. The speedometer consists of seven dials, with the central one colored green, followed by yellow, orange, and red dials. Each dial quantifies the deviation of the measured value from the normative data in terms of mean (μ) and standard deviation (σ), calculated as a z-score based on the healthy control group data. A needle points to a z-score ranging from 0 to ±3: z-score = 0 represents values within ±1σ of the mean, indicating a close match with normative values, while z-score = ±3 indicates values exceeding ±3σ, representing extreme deviations from the normative range. This representation allows for a quick assessment of the players’ velocity and acceleration performances relative to healthy players’ reference values.

#### 2.3.4. ACL-IRD Algorithm—Kinematics

The ACL-IRD algorithm includes an analysis of hip, knee, and ankle joint sagittal plane kinematics comparing the injured and non-injured limb in the ACLR patients. This evaluation is presented through two complementary graphical visualizations: a horizontal bar plot and joint angle curves within the FC window. The horizontal bar plot (Figure 2B) evaluates the asymmetry between the injured and non-injured limb in the sagittal plane for the three joints. Movement asymmetries can lead to inefficiency in movements and increasing risk of primary and secondary ACL injuries [39]. The asymmetry is quantified using an Asymmetry Score (AS), which ranges from 0 to 3 depending on the calculated percentage of asymmetry. Asymmetry is illustrated as a blue vertical line positioned within color-coded intervals that represent increasing levels of asymmetry, from green to red. Deviations from normative values are based on z-score. An asterisk is used to indicate a z-score higher than 3, denoting substantial deviation from the healthy control data for that parameter. On the other hand, joint angle curves provide a comparison between the injured and non-injured limbs, respectively, colored in red and blue, with the normative reference curve represented in gray (mean ± standard deviation) across the FC window (Online Resource S1, page 7, 15).

#### 2.3.5. ACL-IRD Algorithm—Risk Factors

The risk factor section is the key part of the ACL-IRD algorithm. Nine biomechanical risk factors were identified across three main categories: sagittal knee loading (SKL), knee valgus collapse (KVC) and trunk–pelvis Imbalance (TPI). SKL involves hip/knee ratio (HK ratio), hip flexion (HF), knee flexion (KF), and ankle plantarflexion (AF) as factors; KVC includes knee valgus (KV), hip abd/adduction (HA), and hip internal rotation (HI); TPI instead is influenced by trunk ipsilateral bending (TIB) and trunk contralateral bending (TCR) factors. The biomechanical factors belong to previously validated biomechanical patterns associated with non-contact ACL injury profiles [13].

The risk factor section is separated into two critical moments: initial contact (IC) and peak knee flexion (pKF). As stated in prior research, these phases have been central to studies regarding ACL injury prevention and rehabilitation [33,40]. Reference thresholds are calculated from the healthy control group data: a threshold is defined as the mean of the control group ± one standard deviation, with an additional ±5% conservative tolerance factor to account for measurement uncertainty and inter-subject variability (Table 1). A trial was classified as being at overall risk if at least four factors were identified as at risk within the same trial. The thresholds are provided separately for male and female players to avoid sex bias. The quantitative largest deviation from the threshold for each risk factor is presented in a table below the pie chart. The asterisk represents a deviation of more than 100% from the threshold.

**Figure 3:**
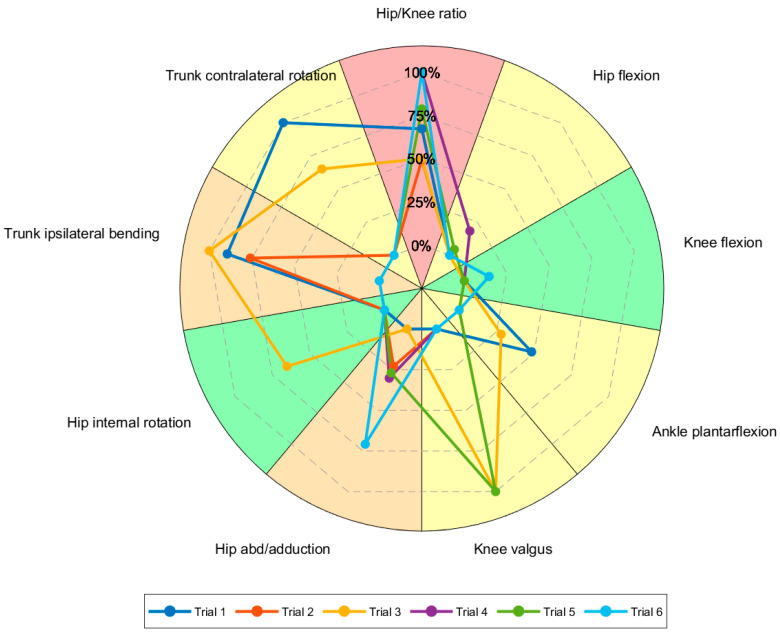
Risk factors visualization. The number of slices in the pie chart corresponds to the number of factors identified as patterns associated with ACL injuries. Slices are color-coded as follows: green if the percentage of trials at risk for the specific factor is lower than 20% (less than 20% of trials are above or below the threshold); yellow if between 20% and 50%; orange if between 50% and 70%; and red if more than 70% of trials are at risk. Legend lists all trials performed by the athlete during one test (both injured and non-injured side). A single line in the spider chart illustrates the percentage deviation of one trial from the normative value across all reported risk factors (four grids scale the percentage deviation from 0% to 100%, with 100% indicating a value that is double or half the threshold).

The visualization combines a color-coded pie chart with an embedded radar chart (Figure 3), allowing for the illustration of both the aggregate risk level for each factor (color-coded in the pie chart, from green to red) and the individual trial deviations (lines of the radar chart; the higher in percentage, the further from normality). Such a visualization allows a comprehensive description of the risk factors section. The risk factors section features a comprehensive dashboard that visually summarizes cumulative and trial-specific risk profiles occurring during the test across the three main categories (SKL, KVC, TPI). Each category is represented by a status circle that turns red when the category is determined to be at risk. Additionally, the risk factor dashboard displays the percentage of trials at risk within their respective categories.

### 2.4. Statistical Analysis

The normal distribution of the data was inspected through the Shapiro–Wilk test and presented as mean ± standard deviation while the categorical data were presented as a percentage over the total or median with interquartile range (IQR) as appropriate. The outcomes of the ACL-IRD algorithm were presented as aggregated results for the whole ACLR players’ cohort and as a single example case. Due to the descriptive nature of the study and the absence of inferential tests, no a priori power analysis was performed. A previous study with coherent methodology and rationale included 28 players considering a power of 0.80 and a partial eta squared of 0.10 to assess differences between different types of change of direction kinematics on the field [22]. Adequateness of the sample size was also confirmed by a scoping review from the literature: according to Preatoni et al., median sample size for studies investigating biomechanics from wearables in sports-related musculoskeletal injuries is 17 healthy athletes (on multisport injuries) [41].

## 3. Results

Overall, 201 trials belonging to the 21 ACLR players were analyzed through the ACL-IRD algorithm. The cohort level results of the ACLR players compared to the healthy controls are presented below. Moreover, each ACLR player received a dedicated report. An example case (ID: ACLR04) is presented to show the typical report functioning.

### 3.1. ACLR Players vs. Healthy Controls

For AGTT, a total of 104 valid trials were performed for the ACLR players (54 trials executed with the injured limb, 50 with the non-injured limb). An average cut angle of 75.0 ± 4.9° was identified. The mean entry velocity was 5.37 ± 0.65 m/s (4/21 ACLR players slower than control group) while the mean exit velocity was 3.13 ± 0.46 m/s (9/21 ACLR players slower than control group). For the FS deceiving action, kinematic data of one player were lost due to error during data collection, and a total of 97 valid trials were recorded for the ACLR players (50 trials executed with injured leg, 47 using the non-injured leg), with an average cut angle of 32.8 ± 6.9°. The mean entry velocity was 5.61 ± 1.19 m/s (16/20 ACLR players slower than control group) while the mean exit velocity was 4.24 ± 1.17 m/s (5/20 ACLR players slower than control group).

**Figure 4:**
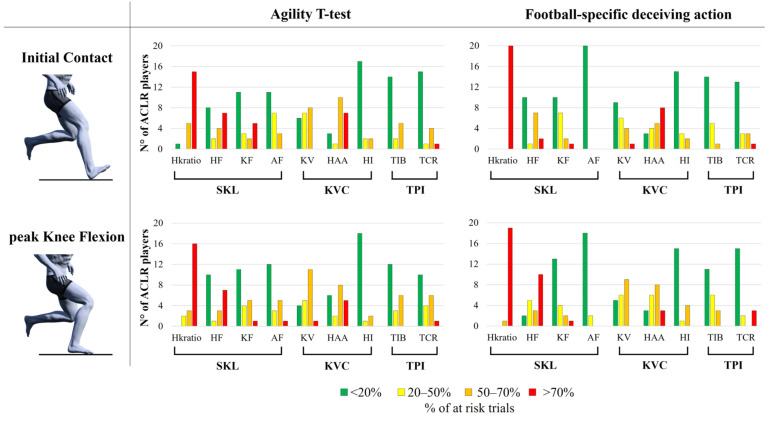
Risk factor detected by the ACL-IRD algorithm at initial contact (top row) and peak knee flexion (bottom row) for the Agility *T*-test (left column) and the football-specific deceiving action (right column). The bar chart illustrates the number of subjects considered at risk (red, orange, and yellow columns) and those who are not (green column) for each specific factor (hip/knee flexion ratio, hip flexion, knee flexion, ankle flexion, knee valgus, hip abduction angle, hip intra-rotation, trunk ipsilateral bending, trunk contralateral rotation). Risk factors are colored according to the percentage of trials considered as at risk for the specific pattern: <20% of trials at risk (green), 20–50% (yellow), 50–70% (orange), and >70% (red).

In AGTT, the ACL-IRD algorithm detected at-risk biomechanics in at least one trial in 21 players out of 21. A total of 35 trials out of 104 (34%) were classified as being at overall risk at IC, with 24 of these trials (69%) performed with the injured limb. Similarly, 44 trials out of 104 (42%) were identified as being at overall risk at pKF, with 26 of these trials (59%) performed with the injured limb. The risk factors associated with the SKL category were found to exceed the normative threshold in the highest number of trials, compared to KVC and TPI categories. In particular, HK ratio was identified as a risk factor in at least half of the trials performed in 20 players (95%) at IC and in all players at pKF (Figure 4, Agility *T*-test). HF was also frequent both at IC (13/21 players) and pKF (11/21 players). The risk factors associated with the KVC category were also considerably frequent, especially regarding KV (15/21 players at IC, 17/21 players at pKF) and HAA (18/21 players at IC, 15/21 players at pKF). The risk factors belonging to the TPI category instead were present in 7/21 players at IC and 9/21 players at pKF for TIB, and in 6/21 players at IC 11/21 players at pKF for TCR.

In FS deceiving action, the ACL-IRD algorithm detected at-risk biomechanics in at least one trial in 19 players out of 20. A total of 28 trials out of 97 (29%) were classified as being at overall risk at IC, with 17 of these trials (61%) performed with the injured limb. Similarly, 46 trials out of 97 (47%) were identified as being at overall risk at pKF, with 24 of these trials (52%) performed with the injured limb. As for the AGTT, the risk factors associated with the SKL category were found to exceed the normative threshold in the highest number of trials, with the HK ratio identified as a risk factor for all players (100%) at both IC and pKF in at least half of the trials (Figure 4, football-specific deceiving action). HF was also frequent at IC (10/20 players), but especially at pKF (18/20 players). The KV at IC (11/20 players) and pKF (15/20 players) and HAA both at IC and pKF (17/20 players) were also frequently detected. Similarly to the AGTT, the TPI category showed a few players exceeding the normative threshold (6/20 and 7/20 players at IC, 9/20 and 5/20 at pKF, for TIB and TCR, respectively).

### 3.2. Example Case: Participant ACLR04

Participant ACLR04 is a competitive male football player (16 years old) who underwent ACLR on the left knee (assessed 38 months after ACLR). He completed both the AGTT and FS deceiving action tests, performing six trials each (three cut maneuvers with the left, injured limb and three with the right, non-injured limb).

The detailed report for this player is presented at Online Resource S1 in the Supplementary Materials.

For the sake of conciseness, only report pages belonging to AGTT (Online Resource S1, page 3–11) were presented. The average cut angle across six trials was 66.0 ± 3.2°. Regarding the player’s performance, in the entry phase of the cut, the peak velocity reached 3.1 m/s, corresponding to a z-score = 0 (aligned with normative values, green dial). In the exit phase of the cut, the peak velocity of 5.3 m/s was slightly below the normative levels (z-score −1 to −2, yellow dial). Peak acceleration in the entry window (Peak Acceleration IN) was 5.7 m/s^2^ (z-score −1 to −2, yellow dial), while deceleration (Min Deceleration IN) in the entry phase (−4.9 m/s^2^) and acceleration (Peak Acceleration OUT) in the exit phase (8.2 m/s^2^) were consistent with normative benchmarks (z-score −1 to +1) (Online Resource S1, page 5–6).

Kinematics asymmetry analysis (Figure 2, Online Resource S1, page 7) revealed slight asymmetries in hip and knee flexion (<20%) and more pronounced asymmetries (>70%) in ankle flexion. Additionally, as indicated by the asterisk, ankle flexion substantially deviated from the normative values.

Regarding the risk factor analysis, 4/9 factors were classified as “at risk” at IC. The four factors were HK ratio, HF (SKL category), HAA (KVC category), and TIB (TPI category), as indicated by the yellow-to-red pies in the pie chart (3/6 to 5/6 trials with the threshold surpassed, Online Resource S1, page 8–9). The radar chart indicated that 3/9 factors (HK ratio, HAA, and AF) deviated more than 75% from the respective threshold in at least one trial. In particular, the table showed that two of these factors (AF and HAA) deviated more than 100% from the threshold (the relative value is presented under the min/max data column). Overall risk was found in 1/6 trials (injured limb), meaning 4/9 factors simultaneously at risk, as shown in the last row of the table. No category turned red at IC (Online Resource S1, page 9), meaning that all the risk factors of a category never simultaneously surpassed the threshold.

At pKF, 7/9 factors were classified as at risk (Online Resource S1 page 10–11). The seven factors were HK ratio, HF, AF (SKL category), HAA, KV (KVC category), and TIB, TCR (TPI category), as indicated by the yellow-to-red pies in the pie chart (Online Resource S1, page 10–11). The radar chart indicated that 4/9 factors (HK ratio, KV, TIB, and TCR) deviated more than 75% from the respective threshold in at least one trial. In particular, the table showed that three of these factors (HK ratio, KV, and TIB) deviated more than 100% from the threshold. Overall risk was found in 3/6 trials, of which two performed with the injured limb and one with the uninjured limb, as shown in the last row of the table. The TPI category turned red at pKF (Online Resource S1, page 11), since all TPI risk factors simultaneously surpassed the threshold in 2/6 trials (33%).

Similar performance, kinematics, and risk factors were reported for the FS deceiving action task, with an increased trend towards the KVC category (Online Resource S1, page 12–19). A final summary of results obtained for the two tests performed by the player is given through two tables presenting risk factors percentages (Online Resource S1, page 20). An empty space for final remarks by the clinician is available in the same last page.

## 4. Discussion

The present study presented a tool to objectively assess the occurrence of a biomechanical risk profile for ACL injury sport-specific testing in the late phase of RTS continuum. The ACL-IRD algorithm detected 30–40% of the trials performed with an overall risk profile, the majority belonged to the ACL-injured limb. This aspect suggests an increased biomechanical risk in the operated leg after RTS, which is also in line with the greater rates of second ACL injury in the ipsilateral than contralateral limb [42]. However, the non-negligible presence of risk trials performed with the non-injured limb highlights the importance of a holistic approach to target overall movement quality in rehabilitation after ACL injury [43]. It should be remarked that the thresholds that define the risk for each factor are derived from a normative population of healthy players (no injuries before and one year after the test) matched for age, level of competition, and sex, evaluated in the identical setting of the ACLR players. More at-risk patterns were recorded at the pKF than IC frame, confirming that the kinematics response during the mid-stance phase elicits the identification of poor neuromuscular control [33,44,45]. HK ratio (the ratio between the median knee flexion angle and the first quartile of the hip flexion angle) emerged as the most prevalent risk factor. Indeed, it was identified in the majority of subjects (>95%) at both IC and pKF (Figure 4). This indicates consistent biomechanical compensation between proximal and distal knee joint segments after ACLR resulting from excessive hip flexion, insufficient knee flexion, or both. The altered hip-to-knee flexion might indicate both quadriceps/hamstring muscular weakness and imbalance [46,47]. The restoration of both anterior and posterior kinetic chain actuators plays a pivotal role in rehabilitation after ACLR, and a documented imbalance and over-strengthening of the quadriceps has already been associated with the risk of secondary injuries [46,48,49]. Knee valgus and hip abduction angle also frequently emerged as risk factors in both movement tasks. The dynamic knee valgus is the most well-known indicator for both primary and secondary ACL injury risk, as widely demonstrated in longitudinal studies [50,51,52,53]. Targeted neuromuscular training commonly includes the avoidance of knee overloading by correcting knee valgus tendency [53,54,55]. However, residual valgus tendency after ACLR and RTS had never been demonstrated during on-field cutting movement tasks before.

The case presented (ACLR04) further explained the potential of the ACL-IRD algorithm. The player exhibited peak velocity comparable to healthy controls in the entry phase of the cut, while peak exit velocity was lower, demonstrating a suboptimal propulsion during the cut movement. Lower velocities during testing might suggest a more cautious movement strategy, which in turn could increase vulnerability to high-intensity movements [22,42]. Following ACL reconstruction, a comprehensive rehabilitation program that prioritizes the redevelopment of speed and acceleration capacities is particularly beneficial for football players. This approach enhances sprint abilities while minimizing the risk of overloading the reconstructed knee ligament [56]. Interestingly, kinematic asymmetries were most pronounced at ankle joint level which also deviated from normative values, suggesting a compensatory strategy in the whole lower limb chain, as previously suggested after ACLR in jump landing tasks [57]. Despite undergoing rehabilitation after ACL reconstruction and being cleared for RTS, the player exhibited typical biomechanical patterns of non-contact ACL injuries. The risk factors arose in both non-specific (AGTT) and football-specific (FS deceiving action) cut maneuvers, with a higher frequency of at-risk trials in the latter [14,58]. Common risk factors between the movements were HK ratio, KV, and HAA, with an increased tendency of the latter in the FS deceiving action. At pKF during AGTT, the TPI category emerged as a risk factor (red button). Though being a conservative metric (all risk factors of one category should surpass the threshold simultaneously in a trial), the traffic light representation (Online Resource S1, page 11) suggests potential areas of improvement for rehabilitation, as for trunk stability in this case [7,14,50,59]. Previous studies already proposed a combined metric to detect knee valgus collapse during cut maneuvers and demonstrated its clinical meaningfulness over a FIFA11+ preventative training protocol [50]. However, this metric has never been incorporated into an algorithm including both frontal, transverse, and sagittal plane biomechanics at both lower limb and trunk level.

Other studies provided and validated scoring systems for the full-body assessment of football players’ movement quality and ACL injury risk. In particular, the Movement Analysis Test (MAT) and the Cutting Movement Assessment Score (CMAS) were designed to inspect poor biomechanics and neuromuscular deficits in a clinical-friendly fashion [33,44]. Despite being excellent solutions for either in-lab and field testing, the two systems are based on 2D video-analysis and cannot account for the systematic assessment of 3D kinematics during multiple cut maneuvers in a football-pitch-size volume of acquisition. Moreover, both systems have a semi-automatic kinematics detection system, while the ACL-IRD algorithm is fully automatic. The systems could be seen as complementary rather than alternative solutions to objectively inspect the quality of players’ motion during the different phases of the RTS continuum [17,19,20].

A similar version of the ACL-IRD algorithm was presented by Di Paolo et al. [13]. The algorithm adopted a risk profile for non-contact ACL injury based on thresholds extracted from the current literature on video analysis of professional football players [14]. The algorithm was tested on two high-dynamics movements collected in-lab (90° change of direction and deceleration tasks) by means of wearable inertial sensors and validated against gold standard marker-based motion capture (the presence of risk profile was associated with higher knee joint moments) [13]. The ACL-IRD algorithm overcame the main limitations of the one from Di Paolo et al. Data were collected on the field instead of the lab, thus increasing ecological validity, and the cut maneuvers were both pre-planned and unplanned instead of pre-planned only, with a real opponent increasing the sport-specificity of the task. The definition of the risk profile was based on thresholds provided by a matched cohort of players instead of literature-based. The population under investigation was indeed of ACLR players instead of healthy players only. Lastly, the presence of a comprehensive report including positional and performance data, alongside kinematic asymmetries, increased the overall validity of the algorithm.

The ACL-IRD algorithm does not embrace artificial intelligence. The need for a multiplanar assessment of ACL injury risk and for the generation of a dedicated reporting system hardly coexists with the classical black box approach of the machine learning algorithms. However, a potential integration of machine learning to further refine the ACL-IRD algorithm should not be excluded. In particular, due to the inherent lack of force calculation on the field, machine learning algorithms could be adopted to provide surrogate joint kinetics and contact forces, as already with promising results by Benjaminse and colleagues [60].

ACL injuries are becoming more frequent, particularly among young athletes, with adolescent females at greater risk. The consequences extend beyond physical aspects, affecting mental health, education, sports participation, and family finances. The present work demonstrates how young players recovering from ACLR could show risk factors for non-contact ACL injury even after clearance for RTS. Making complex biomechanical assessment accessible and understandable for clinicians creates a crucial link between technological offers and clinical demands. By analyzing field movements through a comprehensive, quantitative approach such as the ACL-IRD algorithm it becomes possible for a multidisciplinary team of surgeons, physiotherapists, athletic trainers, and coaches to strengthen, refine, and harmonize targeted training to prevent the risk of second ACL injury occurrence. Lastly, translating such data—typically reserved for technical experts—into interpretable language and visualizations, such as charts and graphs, enables the entire team to support the patient through effective communication and enhances overall RTS management.

This study has several limitations. First, the group of ACLR players assessed with the ACL-IRD is relatively small compared to clinical analysis (*n* < 25). The number of healthy players the normative kinematics was determined from was also relatively small (*n* < 50). However, these sample sizes are either in line or higher than the current literature adopting wearable inertial sensors in sports movement testing for injured and healthy athletes, respectively, [41]. It should also be acknowledged that normative data might not be generalizable to adult and/or professional football players. Similarly, caution should be adopted when generalizing the results of the ACLR players to another cohort undergoing a different ACLR surgical technique. The RTS protocols were not standard for all the ACLR players. This could be considered as a bias towards the adoption of dedicated neuromuscular training to restore proper movement quality and avoid the ACL injury risk factors under investigation. However, such a population represents the standard of care for RTS after ACLR. The assessment of the beneficial effect of specific targeted neuromuscular training on the restoration of adequate (non-risky) biomechanics was out of the scope of the present work but could be an ideal future application of the ACL-IRD algorithm. The algorithm has been developed over two specific change of direction movements, thus not accounting for all possible movements that occur during a football (or other sport) match. The adjustment of the normative thresholds could be provided, including a broader set of cut maneuvers or entire football match biomechanics. The analysis has been performed through one specific inertial sensors’ system. It should be noted that this specific system has been validated for the types and complexity of movements under investigation [30,32] and that the ACL-IRD algorithm is designed to potentially allow input data from any motion capture system with only minimal adjustments required. At the current state, no data are available on second ACL injury in the investigated ACLR cohort. Therefore, it could not be confirmed whether the players identified as being at the highest risk of injury according to the algorithm experienced a second ACL injury. The lack of follow-up data limits the ability to assess the predictive validity of the ACL-IRD tool, and these promising findings should be interpreted cautiously at this stage. Future studies should specifically investigate whether deviations from normative profiles, as identified by the algorithm, are associated with an increased incidence of subsequent ACL injuries. The ACL-IRD algorithm should be tested on large-scale cohorts of prospectively enrolled players to confirm its clinical effectiveness and applicability embracing multiple ACLR surgical techniques, players’ age, level of competitiveness, and RTS protocols.

## 5. Conclusions

The ACL-IRD algorithm is the first clinical-friendly, data-driven tool to assess the ACL injury risk designed for sport-specific biomechanical testing during the RTS continuum after ACLR. The algorithm was able to detect biomechanical risk profiles related to the occurrence of non-contact ACL injury in a cohort of young ACLR players even after clearance for RTS. The potential strengths of this tool rely on the simultaneous assessment of well-known ACL injury risk factors, the objective metrics based on football-specific field data, and real-time feedback to clinicians. Future work will focus on formal external validation of the ACL-IRD tool in larger and more diverse cohorts to confirm its reliability and clinical utility in guiding RTS decisions.

## Figures and Tables

**Table 1:** List of the biomechanical risk factors for ACL injury, divided by gender (male and female), task (AGTT and FS deceiving action), and frame (IC and pKF). AGTT = Agility *T*-test; FS = football-specific; IC = initial contact; pKF = peak knee flexion; HK = hip/knee flexion; HF = hip flexion; KF = knee flexion; AF = ankle flexion; SKL = sagittal knee loading (category); KV = knee valgus; HA = hip abd/adduction; HI = hip rotation; KVC = knee valgus collapse (category); TIB = trunk ipsilateral bending; TCR = trunk contralateral rotation; TPI = trunk–pelvis imbalance (category).

	Male				Female			
	AGTT		FS Deceiving Action		AGTT		FS Deceiving Action	
Risk Factors	IC	pKF	IC	pKF	IC	pKF	IC	pKF
HK ratio	>0.7	>0.4	>0.0	>0.1	>0.6	>0.4	>−0.0	>0.1
HF	>43.5	>46.0	>41.4	>31.0	>43.9	>44.8	>33.8	>26.6
KF	<32.2	<65.0	<26.2	<78.8	<31.7	<64.2	<24.8	<77.9
AF	<−10.7	<−16.6	<−22.8	<−23.8	<−10.9	<−16.5	<−23.1	<−21.5
**SKL** ^a^	=4	=4	=4	=4	=4	=4	=4	=4
KV	>2.3	>1.4	>2.2	>2.4	>2.0	>2.1	>2.1	>3.2
HAbd	>10.3	>10.4	>8.1	>8.0	>9.9	>9.8	>8.0	>7.9
HAdd	<−7.8	<−7.5	<−2.9	<−6.4	<−10.0	<−10.9	<−7.0	<−7.4
HI	>14.5	>10.4	>10.2	>10.2	>13.8	>10.9	>12.4	>10.5
**KVC** ^a^	=3	=3	=3	=3	=3	=3	=3	=3
TIB	>10.3	>8.6	>8.6	>7.1	>8.7	>8.3	>9.7	>8.0
TCR	>7.3	>4.4	>6.5	>5.6	>5.6	>4.9	>7.3	>5.3
**TPI** ^a^	=2	=2	=2	=2	=2	=2	=2	=2
**Overall risk** ^b^	≥4	≥4	≥4	≥4	≥4	≥4	≥4	≥4

^a^ trial is considered at risk in a specific category if all its factors are classified as “at risk”; ^b^ sum of the three main categories’ risk level. The three main risk categories (SKL, KVC, TPI) and the overall risk line are shown in bold in the first column to distinguish category-level and total risk from individual biomechanical factors.

## Data Availability

The data supporting the conclusion of this article and the associated source code will be made available by the authors upon reasonable request.

## Supplementary Materials

The following supporting information can be downloaded at: https://www.mdpi.com/article/10.3390/sports13110391/s1, Online Resource S1. Example of a patient-specific report generated through the ACL-IRD algorithm.

## Abbreviations

The following abbreviations are used in this manuscript:

ACL	Anterior Cruciate Ligament
ACL-IRD	Anterior Cruciate Ligament Injury Risk Profile Detection
RTS	Return to Sport
ACLR	ACL-reconstructed
AGTT	Agility *T*-test
COD	Change of Direction
FC	Foot Contact
SKL	Sagittal Knee Loading
KVC	Knee Valgus Collapse
TPI	Trunk/Pelvis Imbalance
HK ratio	Hip/Knee ratio
HF	Hip Flection
KF	Knee flexion
AF	Ankle Flexion
KV	Knee Valgus
HA	Hip Abd/adduction
HI	Hip Internal Rotation
TIB	Trunk Ipsilateral Bending
TCR	Trunk Controlateral Bending
IC	Initial Contact
pKF	Peak Knee Flexion
IQR	Interquartile Range
