# Supplementary material for: Testing ACL-Reconstructed Football Players on the Field: An Algorithm to Assess Cutting Biomechanics Injury Risk Through Wearable Sensors
Source: Sports (Basel). 2025 Nov 5;13(11):391. doi: 10.3390/sports13110391 (PMC12655897; doi:10.3390/sports13110391)
Supplement: Supplementary file 1 [file sports-13-00391-s001.zip › sports-3908651-supplementary.pdf]

**Online Resource S1**. Example of a patient-specific report generated through the ACL-IRD algorithm. The report is automatically extracted from the custom Matlab script to a pdf format and provided to the clinicians.

---

Name:
Age: 16
Sex: Male
Sport: Football
Injured Leg: Left
Participant ID: ACLR04
Number of trials: 6
Report Date: 28 Jan 2023
Test Date: 13 Sep 2022

# Results Report ACL-IRD Field Testing

This document presents the results of the Agility T-test and the Unplanned Football-Specific deceiving action test performed by the participant.
The analysis begins with an overview of parameters related to the cut angle and overall performance. This is followed by a section dedicated to kinematics, where an asymmetry graph and joint flexion plots provide insights into the participant's specific movement patterns. Subsequently, the document focuses on identifying at-risk parameters during key moments of a change of direction. At initial foot contact (IC) and peak knee flexion (pKF), a chart and a table highlight factors that may contribute to an increased risk of injury. The risk analysis is based on three main categories: sagittal knee loading, knee valgus collapse, and trunk/pelvis imbalance. In the concluding section, the document summarizes the findings in a table.
A trial is included in the overall risk model if at least four out of nine parameters are identified as risk factors. Furthermore, a trial is considered at risk for a specific category if all parameters within that category exceed their respective thresholds.

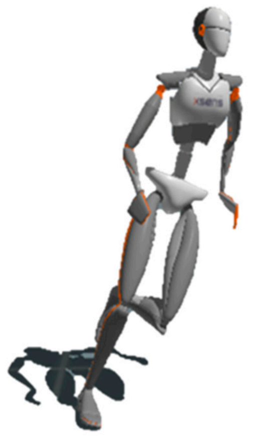

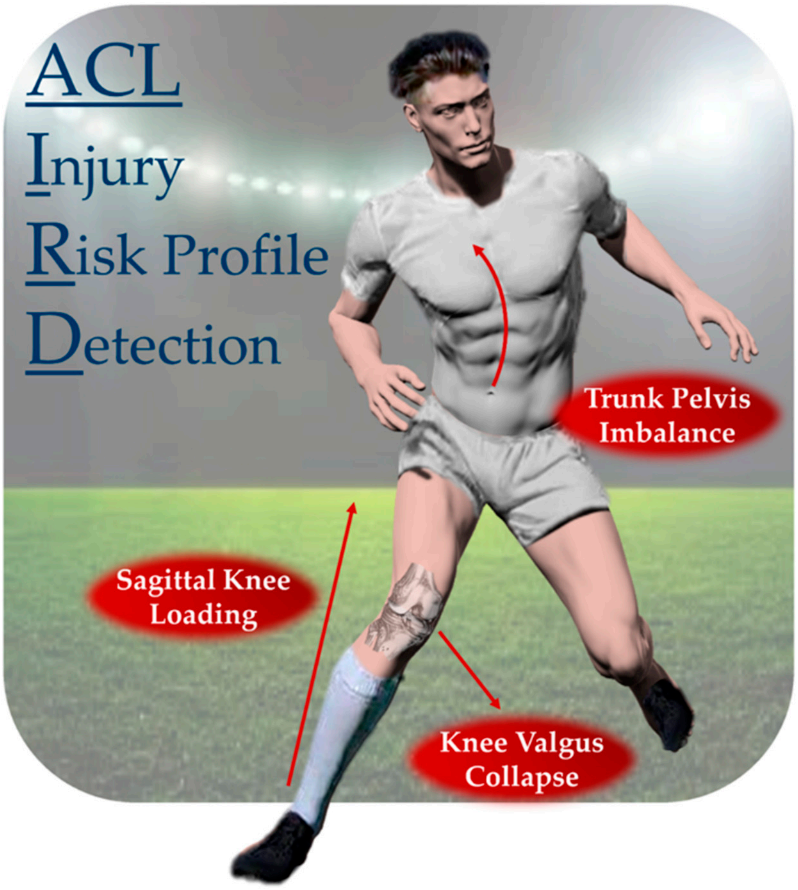
ACL
Injury
Risk Profile
Detection
Trunk Pelvis Imbalance
Sagittal Knee Loading
Knee Valgus Collapse

# Agility T-test

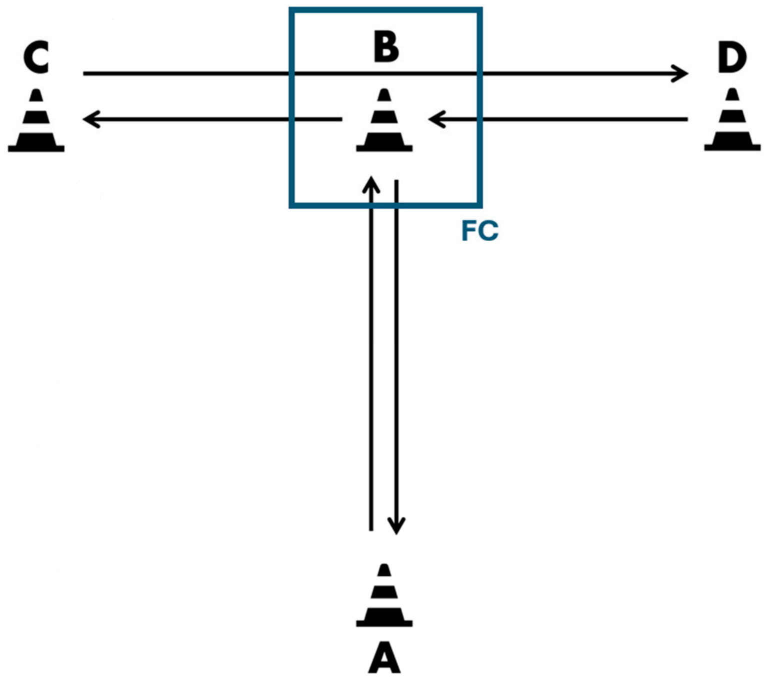


The T-test is an Agility T-test widely used in sports to assess an athlete's ability to change direction, by evaluating acceleration, deceleration and speed.
The test consists of 5 sequential phases: a straight-line sprint from a standing start (A) to a central cone (B), a pre-determined lateral shuffle to one side (D or C), a lateral shuffle to the opposite side (C or D), a return shuffle to the central cone (B), and a final backward sprint to the starting position (A).
The aim here was to make this test the quickest possible while always facing the same direction as the first sprint.

# Cut Angle

The cut angle is the angle between the initial direction of movement (A → B) and the new direction after the change (B → D).
The red area represents the Foot Contact (FC) window where the angle is executed.

| N | N | N | I | I | I | Avg |
|---|---|---|---|---|---|---|
| 66.7 ° | 62.4 ° | 64.1 ° | 68.3 ° | 62.9 ° | 71.4 ° | 66.0 ° |

# Performance Analysis

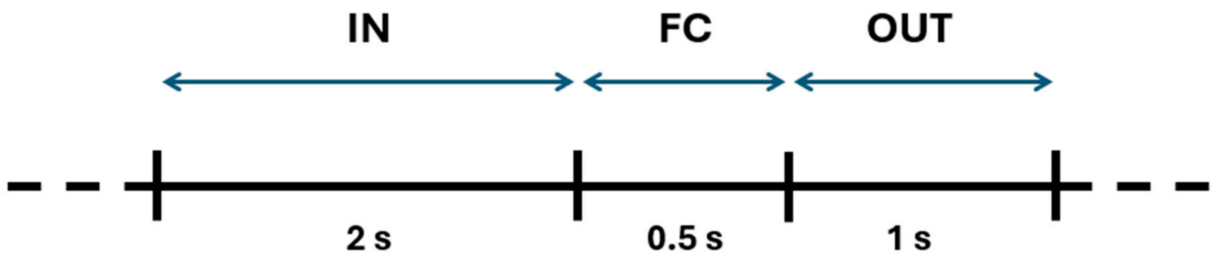


Speed and acceleration are evaluated in an entry window (IN), 2 seconds before FC, and an exit window (OUT), 1 second after FC.
Metrics are displayed on speedometers, with green indicating performance that aligns closely with the normative group, and red showing the greatest deviation from the normative values.

## Velocity

Peak Velocity IN: Highest speed during the approach phase.
Peak Velocity OUT: Top speed during the re-acceleration phase.

**Peak Velocity IN**

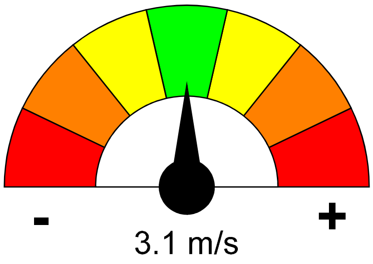


**Peak Velocity OUT**

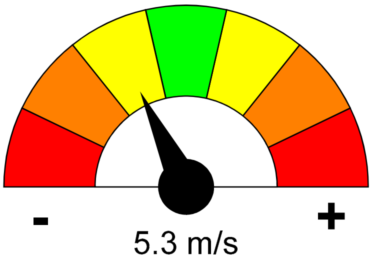


## Acceleration and Deceleration

Peak Acceleration IN: Maximum acceleration toward the change of direction, prior to braking.
Min Deceleration IN: Maximum speed reduction (braking intensity).
Peak Acceleration OUT: Maximum acceleration into the new direction.

**Peak Acceleration IN**

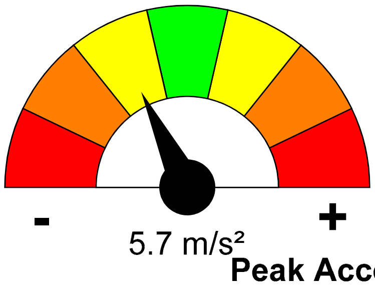


**Min Deceleration IN**

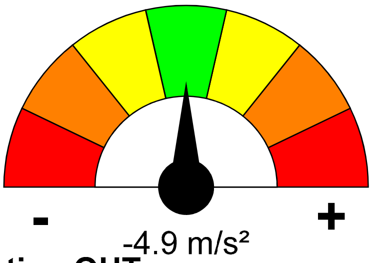


**Peak Acceleration OUT**

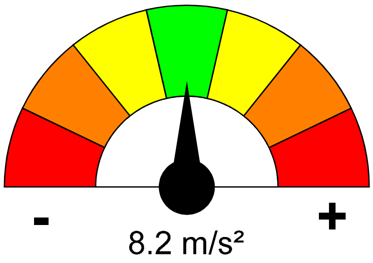

# Asymmetry

Asymmetry is the average difference in joint motion (flexion and extension) between the injured and non-injured limbs.
The graph highlights potential imbalances, with the blue line indicating the side with greater joint movement.

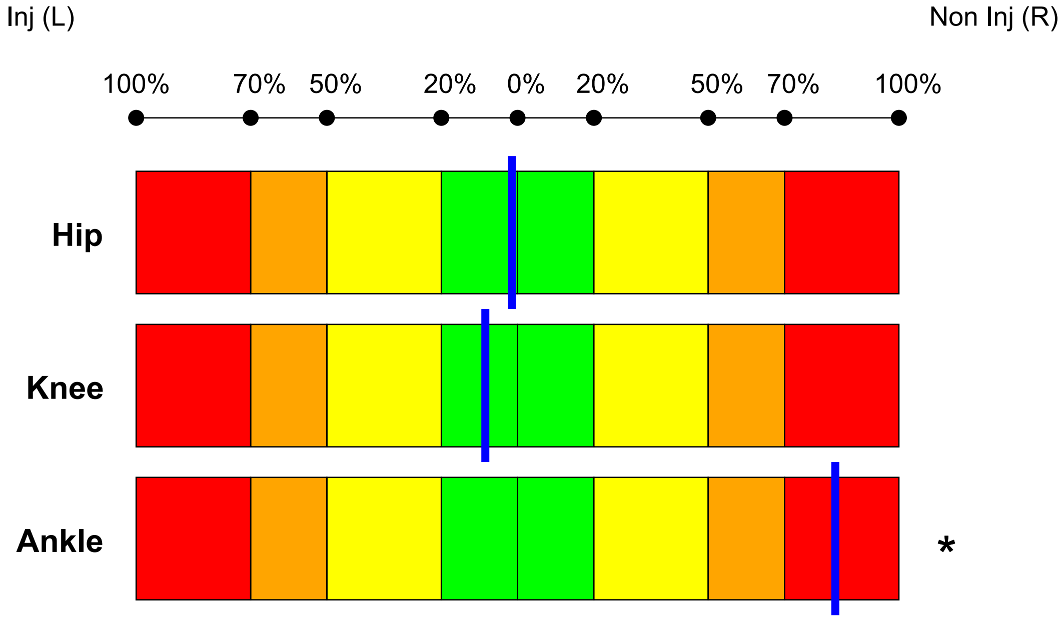


Graphs represent joint flexion during the FC window.

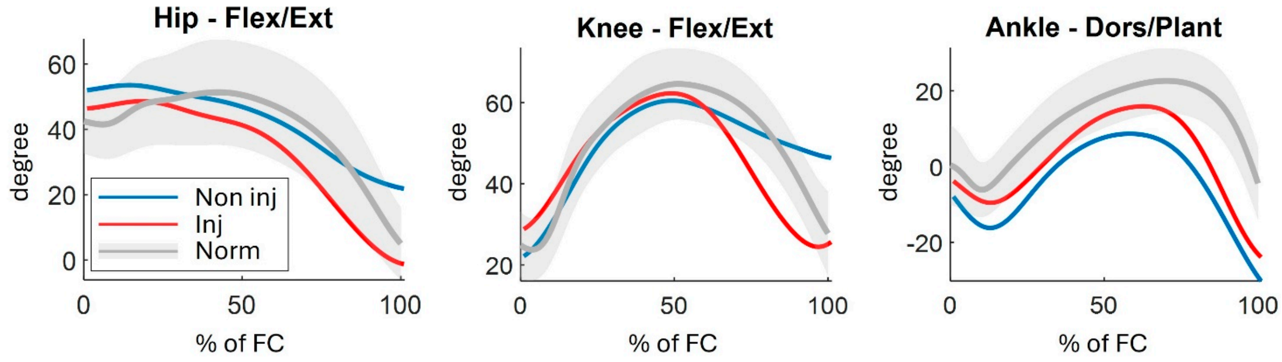

# Risk Factor (ACL-IRD)

At initial foot contact (IC) and peak knee flexion (pKF), three main categories are analysed: Sagittal Knee Loading, Knee Valgus Collapse, Trunk/Pelvis Imbalance, each divided into specific parameters listed in the table below.
All parameters are assessed to determine a risk level, based on whether they exceed defined thresholds. The chart below used a color-coding system to visualize the risk level.
Each slice of the pie, representing a single parameter, is coloured based on the percentage of trials at risk for that specific factor. Inside each slice, dots represent individual trials, with their distance from the centre indicating the percentage by which their value exceeds the threshold.

# Risk Factor - Initial foot Contact (IC)

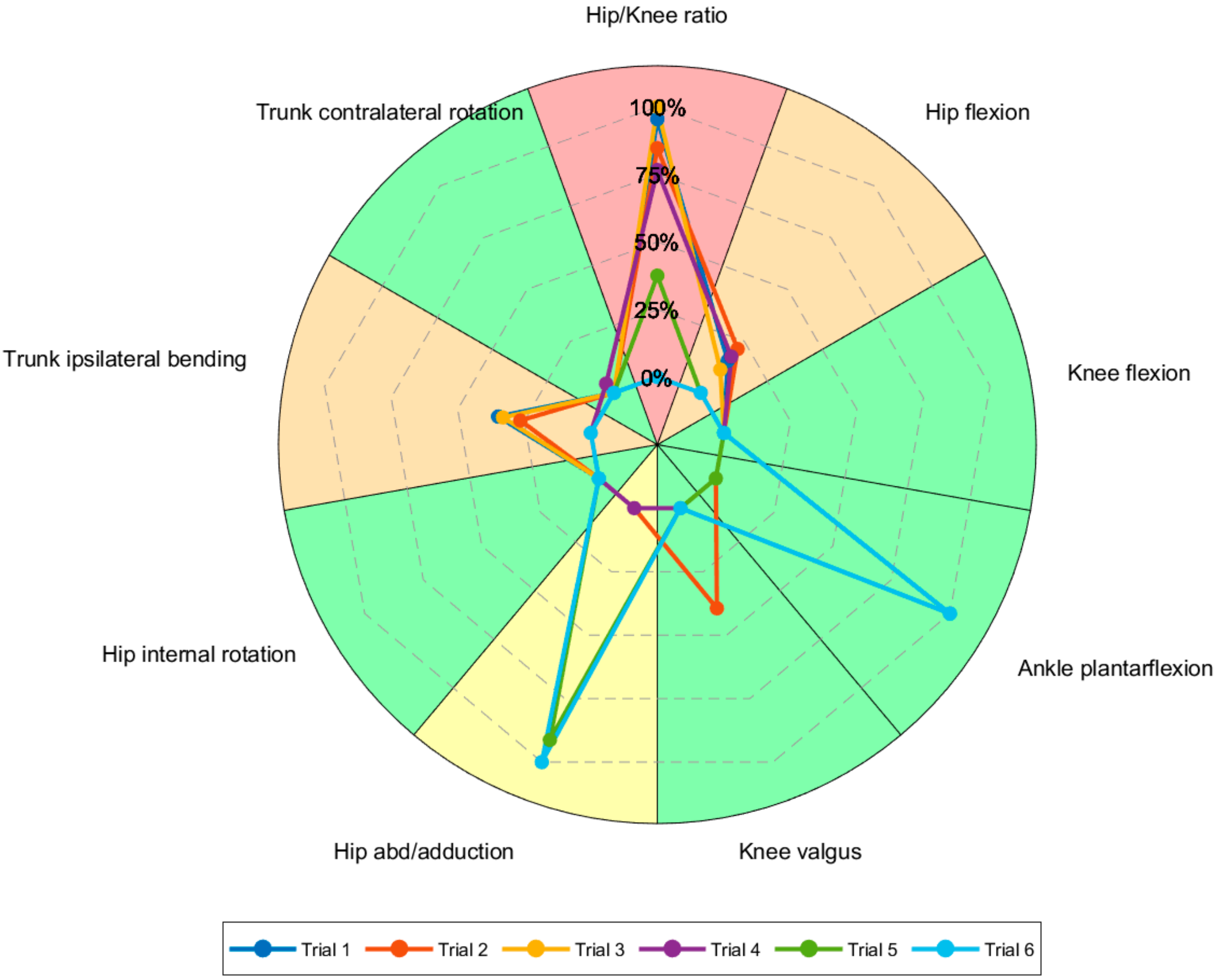


| Risk Factors | Threshold | min/max data | I | I | I | N | N | N |
|---|---|---|---|---|---|---|---|---|
| **Sagittal Knee Loading** | **=4** | | **2** | **2** | **2** | **2** | **1** | **1** |
| Hip/knee ratio | >0.66 | 0.73 | 1 | 1 | 1 | 1 | 1 | 0 |
| Hip flexion | >43.47 | 51.09 | 1 | 1 | 1 | 1 | 0 | 0 |
| Knee flexion | <32.21 | | 0 | 0 | 0 | 0 | 0 | 0 |

| | | | | | | | | |
|---|---|---|---|---|---|---|---|---|
| Ankle plantarflexion | <-10.71 | -25.05* | 0 | 0 | 0 | 0 | 0 | 1 |
| **Knee Valgus Collapse** | **=3** | | **0** | **1** | **0** | **0** | **1** | **1** |
| Knee valgus | >2.31 | 3.22 | 0 | 1 | 0 | 0 | 0 | 0 |
| Hip abd/adduction | <-7.77;>10.29 | -16.77* | 0 | 0 | 0 | 0 | 1 | 1 |
| Hip internal rotation | >14.49 | | 0 | 0 | 0 | 0 | 0 | 0 |
| **Trunk/Pelvis Imbalance** | **=2** | | **1** | **1** | **1** | **1** | **0** | **0** |
| Trunk ipsilateral bending | >10.29 | 13.69 | 1 | 1 | 1 | 0 | 0 | 0 |
| Trunk contralateral rotation | >7.25 | 7.57 | 0 | 0 | 0 | 1 | 0 | 0 |
| **Total** | **=4** | | **3** | **4** | **3** | **3** | **2** | **2** |

* The value is double or half the threshold

I = Injured limb; N = Non-injured limb

The risk level during IC across the three main categories (sagittal knee loading, knee valgus collapse, trunk/pelvis imbalance) is summarized.
A red button determines an at-risk category and shows the percentage of trials at risk for it.

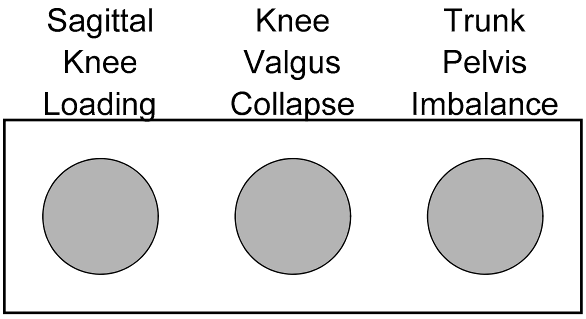

# Risk Factor - Peak knee flexion (pKF)

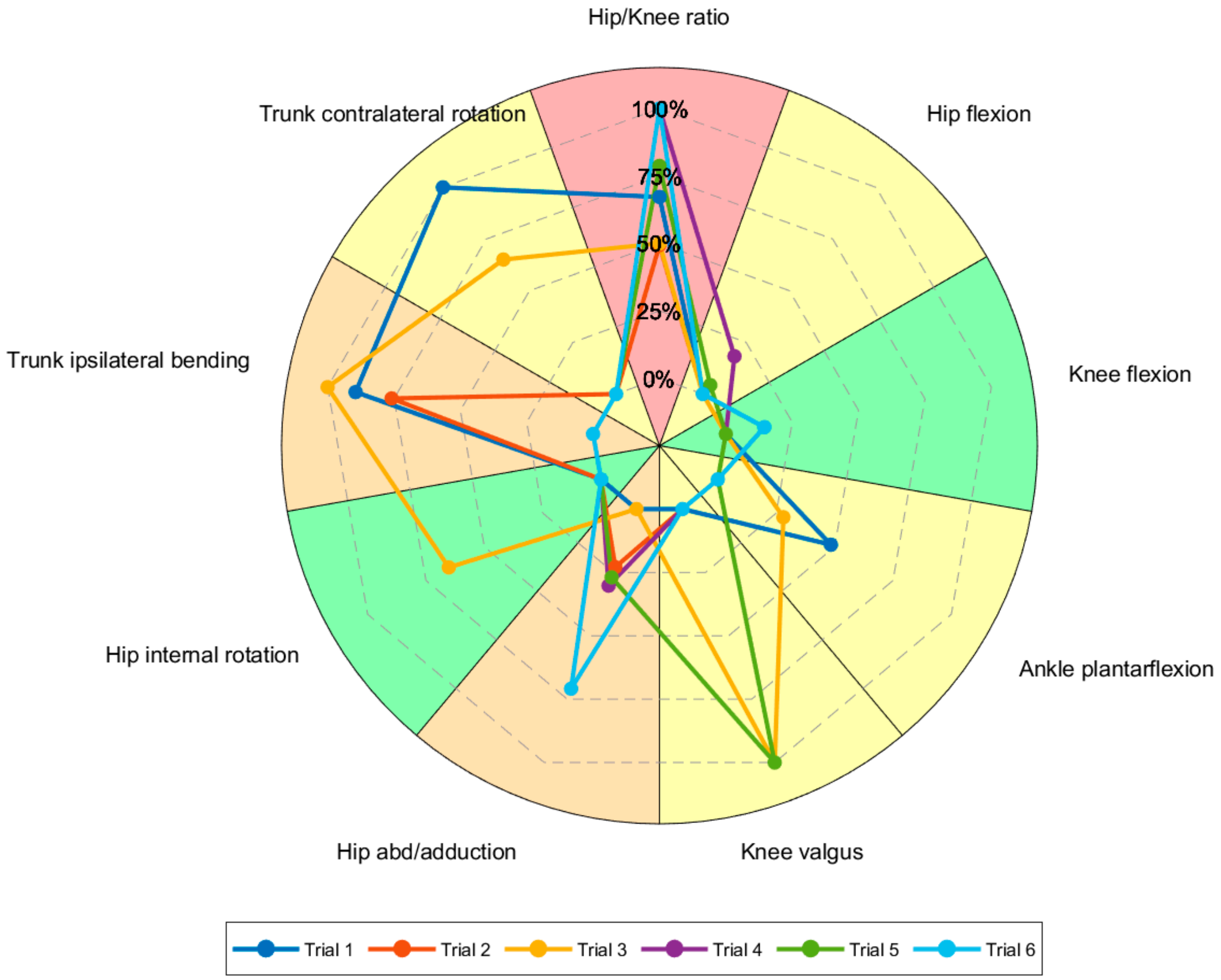

| Risk Factors | Threshold | min/max data | I | I | I | N | N | N |
|---|---|---|---|---|---|---|---|---|
| **Sagittal Knee Loading** | **=4** | | **2** | **1** | **2** | **2** | **2** | **2** |
| Hip/knee ratio | >0.36 | 0.73* | 1 | 1 | 1 | 1 | 1 | 1 |
| Hip flexion | >45.99 | 47.97 | 0 | 0 | 0 | 1 | 1 | 0 |
| Knee flexion | <64.98 | 55.56 | 0 | 0 | 0 | 0 | 0 | 1 |
| Ankle plantarflexion | <-16.59 | -21.25 | 1 | 0 | 1 | 0 | 0 | 0 |
| **Knee Valgus Collapse** | **=3** | | **0** | **1** | **2** | **1** | **2** | **1** |
| Knee valgus | >1.37 | 6.12* | 0 | 0 | 1 | 0 | 1 | 0 |
| Hip abd/adduction | <-7.46;>10.40 | -12.75<br>12.78 | 0 | 1 | 0 | 1 | 1 | 1 |
| Hip internal rotation | >10.40 | 17.19 | 0 | 0 | 1 | 0 | 0 | 0 |
| **Trunk/Pelvis Imbalance** | **=2** | | **2** | **1** | **2** | **0** | **0** | **0** |
| Trunk ipsilateral bending | >8.61 | 19.36* | 1 | 1 | 1 | 0 | 0 | 0 |
| Trunk contralateral rotation | >4.41 | 7.28 | 1 | 0 | 1 | 0 | 0 | 0 |
| **Total** | **=4** | | **4** | **3** | **6** | **3** | **4** | **3** |

* The value is double or half the threshold
I = Injured limb; N = Non-injured limb

The risk level during pKF across the three main categories (sagittal knee loading, knee valgus collapse, trunk/pelvis imbalance) is summarized.
A red button determines an at-risk category and shows the percentage of trials at risk for it.

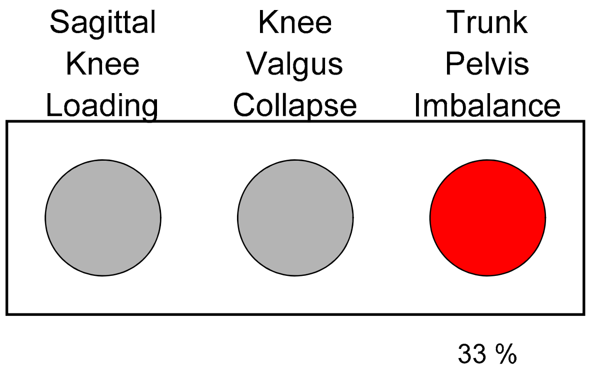

# Unplanned Football-Specific deceiving action

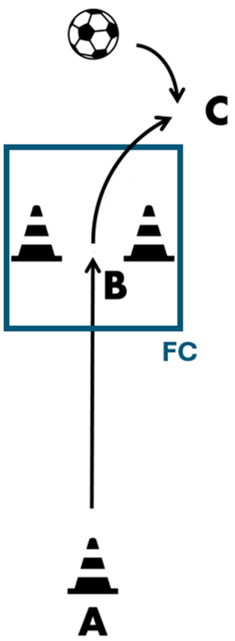


The Unplanned Football-Specific deceiving action test is used to determine the adaptability and the speed of the participant.
For this test, the participant was asked to run in a straight line from the point A to the point B, then an opponent made a deceiving action with the ball in possess and the participant changed his/her direction towards him (C), simulating a pressing situation. Thus, the player makes an unexpected change of direction. Thus, the player makes an unexpected change of direction.

# Cut Angle

The cut angle is the angle between the initial direction of movement (A → B) and the new direction after the change (B → D).
The red area represents the Foot Contact (FC) window where the angle is executed.

| N | N | N | I | I | I | Avg |
|---|---|---|---|---|---|---|
| 31.4 ° | 41.4 ° | 29.3 ° | 42.6 ° | 37.9 ° | 39.5 ° | 37.0 ° |

# Performance Analysis

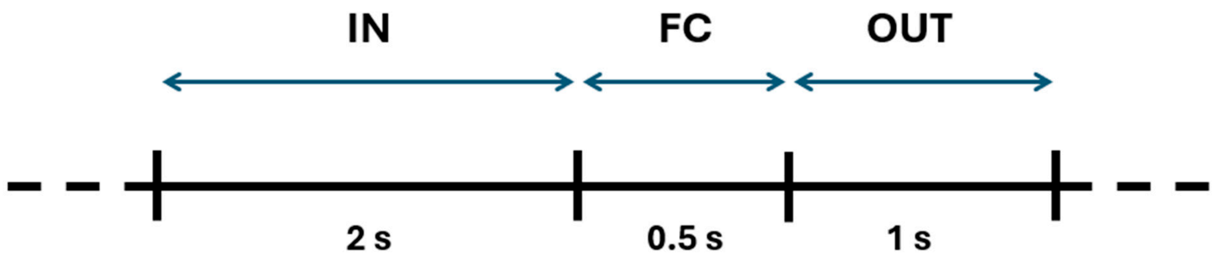


Speed and acceleration are evaluated in an entry window (IN), 2 seconds before FC, and an exit window (OUT), 1 second after FC.
Metrics are displayed on speedometers, with green indicating performance that aligns closely with the normative group, and red showing the greatest deviation from the normative values.

## Velocity

Peak Velocity IN: Highest speed during the approach phase.
Peak Velocity OUT: Top speed during the re-acceleration phase.

**Peak Velocity IN**

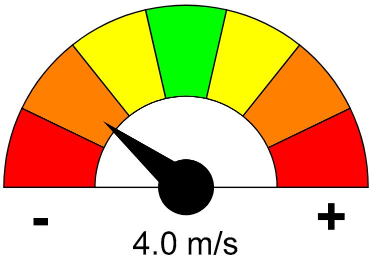


**Peak Velocity OUT**

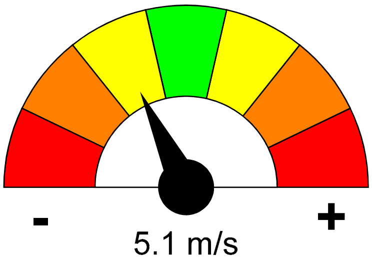


## Acceleration and Deceleration

Peak Acceleration IN: Maximum acceleration toward the change of direction, prior to braking.
Min Deceleration IN: Maximum speed reduction (braking intensity).
Peak Acceleration OUT: Maximum acceleration into the new direction.

**Peak Acceleration IN**

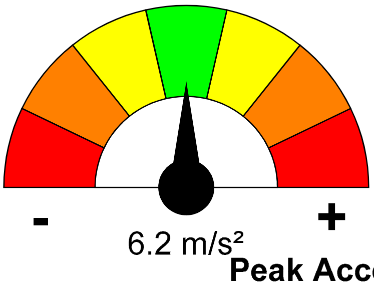


**Min Deceleration IN**

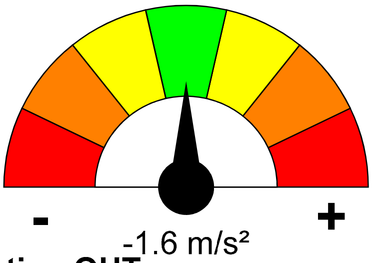


**Peak Acceleration OUT**

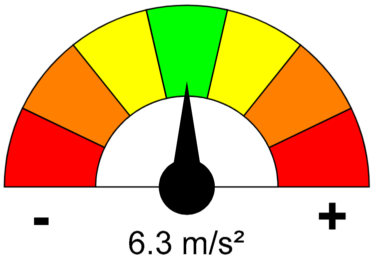

# Asymmetry

Asymmetry is the average difference in joint motion (flexion and extension) between the injured and non-injured limbs.
The graph highlights potential imbalances, with the blue line indicating the side with greater joint movement.

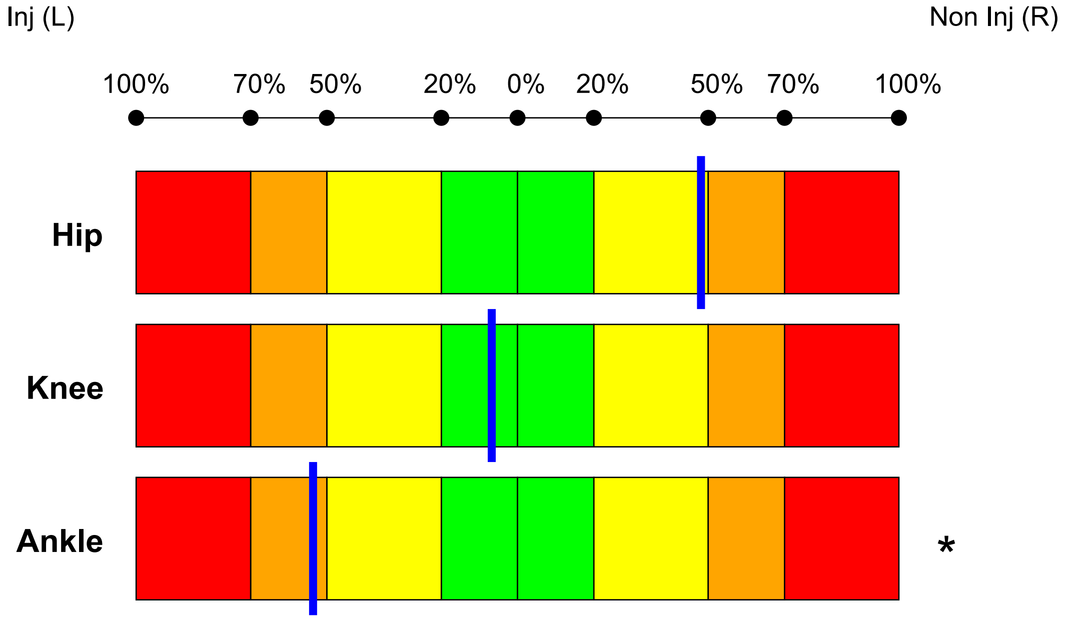


Graphs represent joint flexion during the FC window.

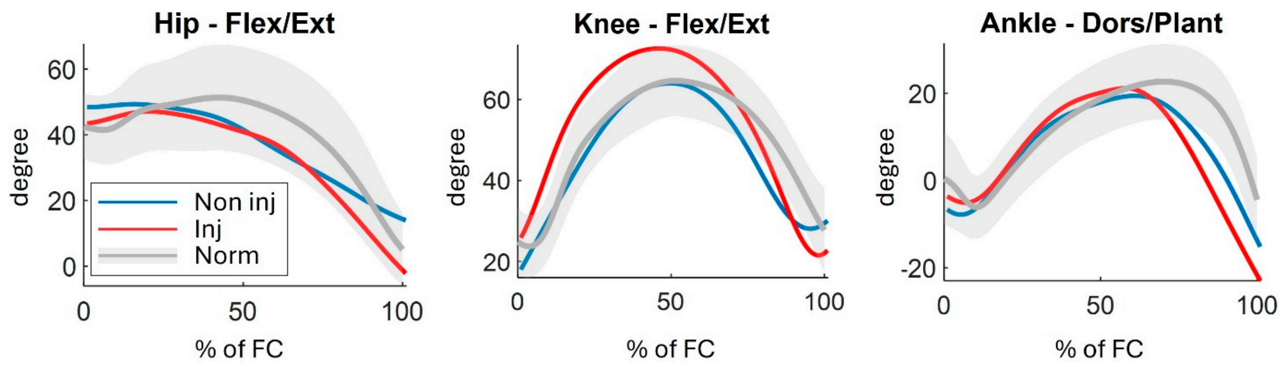

# Risk Factor (ACL-IRD)

At initial foot contact (IC) and peak knee flexion (pKF), three main categories are analysed: Sagittal Knee Loading, Knee Valgus Collapse, Trunk/Pelvis Imbalance, each divided into specific parameters listed in the table below.
All parameters are assessed to determine a risk level, based on whether they exceed defined thresholds. The chart below used a color-coding system to visualize the risk level.
Each slice of the pie, representing a single parameter, is coloured based on the percentage of trials at risk for that specific factor. Inside each slice, dots represent individual trials, with their distance from the centre indicating the percentage by which their value exceeds the threshold.

# Risk Factor - Initial foot Contact (IC)

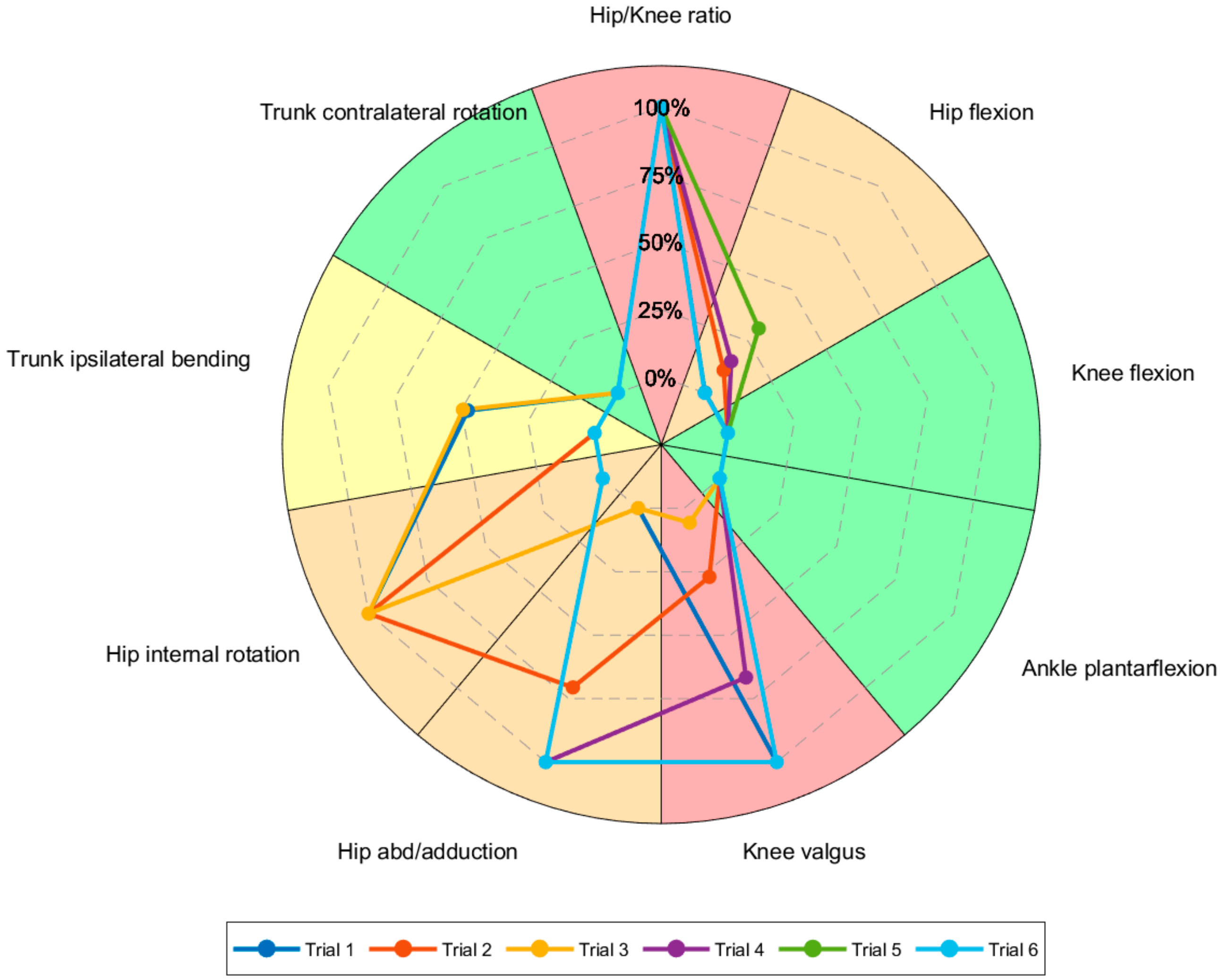


| Risk Factors | Threshold | min/max data | I | I | I | N | N | N |
|---|---|---|---|---|---|---|---|---|
| **Sagittal Knee Loading** | **=4** | | **2** | **2** | **1** | **2** | **2** | **1** |
| Hip/knee ratio | >0.02 | 0.57* | 1 | 1 | 1 | 1 | 1 | 1 |
| Hip flexion | >41.37 | 54.24 | 1 | 1 | 0 | 1 | 1 | 0 |
| Knee flexion | <26.22 | | 0 | 0 | 0 | 0 | 0 | 0 |

| | | | | | | | | |
|---|---|---|---|---|---|---|---|---|
| Ankle plantarflexion | <-22.79 | | 0 | 0 | 0 | 0 | 0 | 0 |
| **Knee Valgus Collapse** | **=3** | | **2** | **3** | **2** | **2** | **2** | **2** |
| Knee valgus | >2.21 | 6.45* | 1 | 1 | 1 | 1 | 1 | 1 |
| Hip abd/adduction | <-2.94;>8.09 | -6.26* | 0 | 1 | 0 | 1 | 1 | 1 |
| Hip internal rotation | >10.19 | 26.06* | 1 | 1 | 1 | 0 | 0 | 0 |
| **Trunk/Pelvis Imbalance** | **=2** | | **1** | **0** | **1** | **0** | **0** | **0** |
| Trunk ipsilateral bending | >8.61 | 12.87 | 1 | 0 | 1 | 0 | 0 | 0 |
| Trunk contralateral rotation | >6.51 | | 0 | 0 | 0 | 0 | 0 | 0 |
| **Total** | **=4** | | **5** | **5** | **4** | **4** | **4** | **3** |

* The value is double or half the threshold
I = Injured limb; N = Non-injured limb

The risk level during IC across the three main categories (sagittal knee loading, knee valgus collapse, trunk/pelvis imbalance) is summarized.
A red button determines an at-risk category and shows the percentage of trials at risk for it.

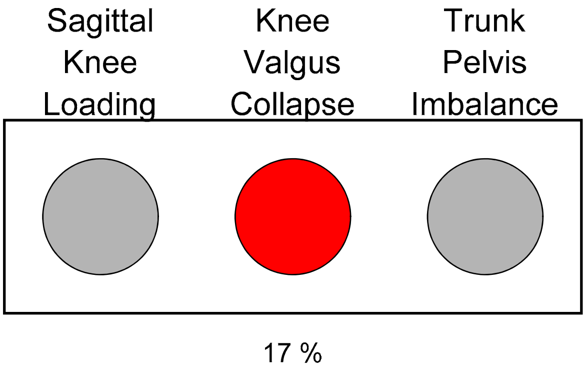

# Risk Factor - Peak knee flexion (pKF)

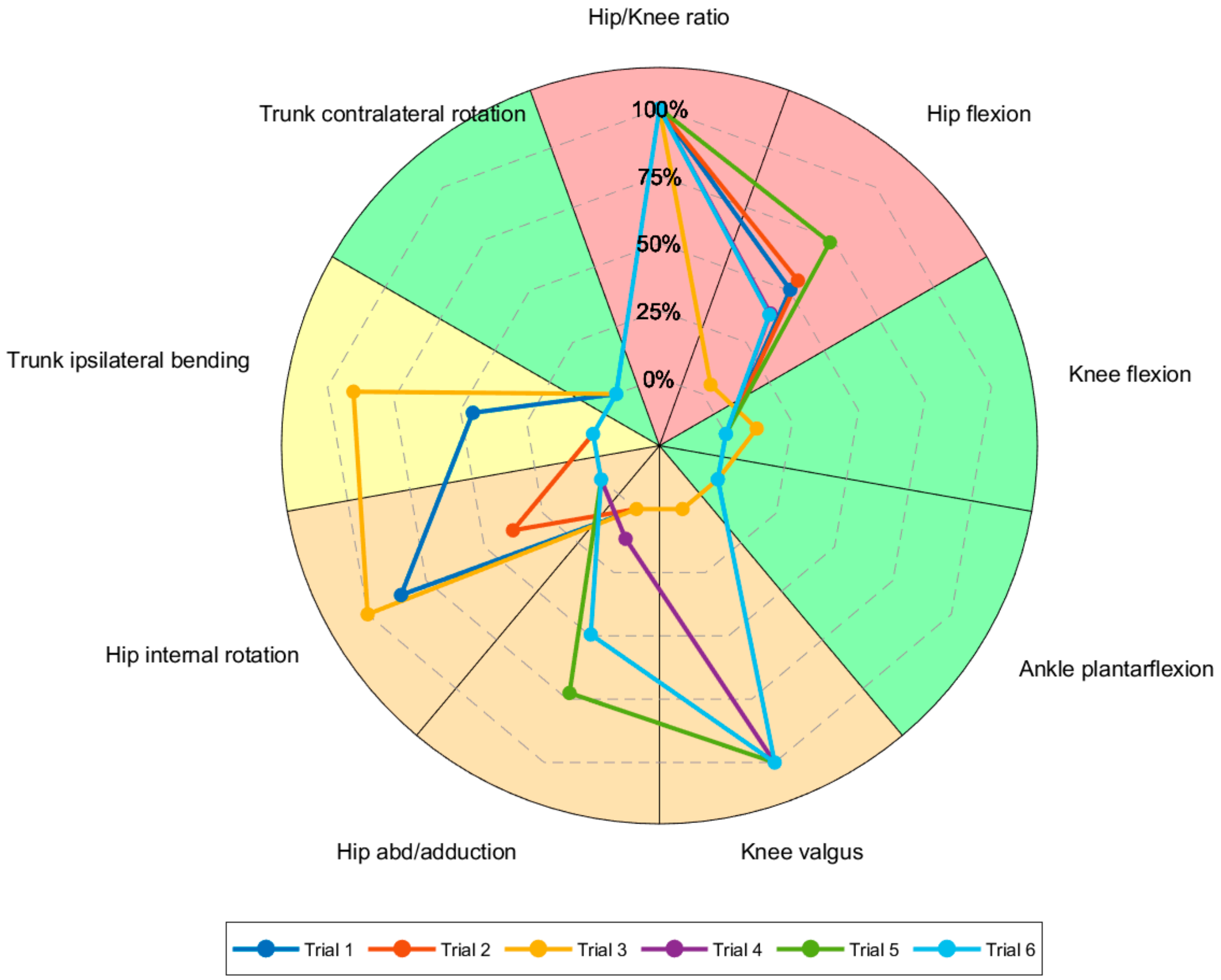

| Risk Factors | Threshold | min/max data | I | I | I | N | N | N |
|---|---|---|---|---|---|---|---|---|
| **Sagittal Knee Loading** | **=4** | | **2** | **2** | **3** | **2** | **2** | **2** |
| Hip/knee ratio | >0.10 | 0.51* | 1 | 1 | 1 | 1 | 1 | 1 |
| Hip flexion | >30.98 | 42.87 | 1 | 1 | 1 | 1 | 1 | 1 |
| Knee flexion | <78.76 | 69.6 | 0 | 0 | 1 | 0 | 0 | 0 |
| Ankle plantarflexion | <-23.84 | | 0 | 0 | 0 | 0 | 0 | 0 |
| **Knee Valgus Collapse** | **=3** | | **1** | **1** | **1** | **2** | **2** | **2** |
| Knee valgus | >2.42 | 8.15* | 0 | 0 | 0 | 1 | 1 | 1 |
| Hip abd/adduction | <-6.41;>7.98 | -9.58 | 0 | 0 | 0 | 1 | 1 | 1 |
| Hip internal rotation | >10.19 | 25.08* | 1 | 1 | 1 | 0 | 0 | 0 |
| **Trunk/Pelvis Imbalance** | **=2** | | **1** | **0** | **1** | **0** | **0** | **0** |
| Trunk ipsilateral bending | >7.14 | 13.59 | 1 | 0 | 1 | 0 | 0 | 0 |
| Trunk contralateral rotation | >5.57 | | 0 | 0 | 0 | 0 | 0 | 0 |
| **Total** | **=4** | | **4** | **3** | **5** | **4** | **4** | **4** |

* The value is double or half the threshold
I = Injured limb; N = Non-injured limb

The risk level during pKF across the three main categories (sagittal knee loading, knee valgus collapse, trunk/pelvis imbalance) is summarized.
A red button determines an at-risk category and shows the percentage of trials at risk for it.

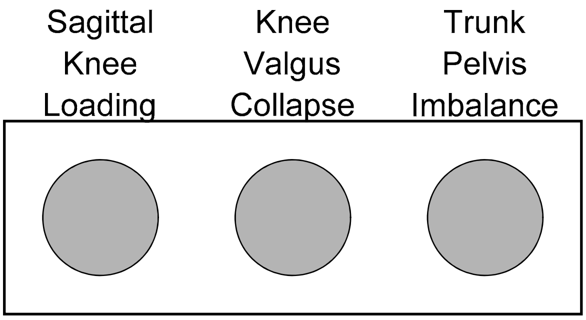

# Summary

## Agility T-test

| Limb | Total trials | IC risk trials | IC % | pKF risk trials | pKF % |
|---|---|---|---|---|---|
| Injured | 3 | 1 | 33.33 | 2 | 66.67 |
| Non Injured | 3 | 0 | 0 | 1 | 33.33 |
| **Total** | **6** | **1** | **16.67** | **3** | **50** |

## Unplanned Football-Specific deceiving action

| Limb | Total trials | IC risk trials | IC % | pKF risk trials | pKF % |
|---|---|---|---|---|---|
| Injured | 3 | 3 | 100.00 | 2 | 66.67 |
| Non Injured | 3 | 2 | 66.67 | 3 | 100 |
| **Total** | **6** | **5** | **83.33** | **5** | **83.33** |

## Note
